# Analysis of the 2020 Taal Volcano tephra fall deposits from crowdsourced information and field data

**DOI:** 10.1007/s00445-022-01534-y

**Published:** 2022-03-02

**Authors:** M. I. R. Balangue-Tarriela, A. M. F. Lagmay, D. M. Sarmiento, J. Vasquez, M. C. Baldago, R. Ybañez, A. A. Ybañez, J. R. Trinidad, S. Thivet, L. Gurioli, B. Van Wyk de Vries, M. Aurelio, D. J. Rafael, A. Bermas, J. A. Escudero

**Affiliations:** 1 UP National Institute of Geological Sciences, College of Science, University of the Philippines, Diliman, Quezon City, Metro Manila Philippines; 2 UP Resilience Institute and NOAH Center, University of the Philippines, Diliman, Quezon City, Metro Manila Philippines; 3 Laboratoire Magmas et Volcans, Université Clermont Auvergne, CNRS, IRD, OPGC, F-63000 Clermont-Ferrand, France

**Keywords:** Taal 2020, Crowdsourced, Tephra fall

## Abstract

After 43 years of dormancy, Taal Volcano violently erupted in January 2020 forming a towering eruption plume. The fall deposits covered an area of 8605 km^2^, which includes Metro Manila of the National Capital Region of the Philippines. The tephra fall caused damage to crops, traffic congestion, roof collapse, and changes in air quality in the affected areas. In a tropical region where heavy rains are frequent, immediate collection of data is crucial in order to preserve the tephra fall deposit record, which is readily washed away by surface water runoff and prevailing winds. Crowdsourcing, field surveys, and laboratory analysis of the tephra fall deposits were conducted to document and characterize the tephra fall deposits of the 2020 Taal Volcano eruption and their impacts. Results show that the tephra fall deposit thins downwind exponentially with a thickness half distance of about 1.40 km and 9.49 km for the proximal and distal exponential segments, respectively. The total calculated volume of erupted fallout deposit is 0.057 km^3^, 0.042 km^3^, or 0.090 km^3^ using the exponential, power-law, and Weibull models, respectively, and all translate to a VEI of 3. However, using a probabilistic approach (Weibull method) with 90% confidence interval, the volume estimate is as high as 0.097 km^3^. With the addition of the base surge deposits amounting to 0.019 km^3^, the volume translates to a VEI of 4, consistent with the classification for the observed height and umbrella radius of the 2020 main eruption plume. VEI 4 is also consistent with the calculated median eruption plume height of 17.8 km and sub-plinian classification based on combined analysis of isopleth and isopach data. Phreatomagmatic activity originated from a vent located in Taal Volcano’s Main Crater Lake (MCL), which contained 42 million m^3^ of water. This eruptive style is further supported by the characteristics of the ash grain components of the distal 12 January 2020 tephra fall deposits, consisting dominantly of andesitic vitric fragments (83–90%). Other components of the fall deposits are lithic (7–11%) and crystal (less than 6%) grains. Further textural and geochemical analysis of these tephra fall deposits contributes to better understand the volcanic processes that occurred at Taal Volcano, one of the 16 Decade Volcanoes identified by the International Association of Volcanology and Chemistry of the Earth’s Interior (IAVCEI) because of its destructive nature and proximity to densely populated areas. The crowdsourcing initiative provided a significant portion of the data used for this study while at the same time educating and empowering the community to build resilience.

**Supplementary Information:**

The online version contains supplementary material available at 10.1007/s00445-022-01534-y.

## Introduction

After 43 years of repose, Taal Volcano violently erupted on 12 January 2020, forming a plume that reached 17–21 km above sea level (PTCC 2020; Perttu et al. 2020; Bachmeier 2020). The eruption started at around 1 p.m. (all times described in this manuscript are local times, which correspond to UTC+ 8) as a series of phreatic explosions involving heated ground water or lake water. The volcano Alert Level was raised to 2 at 2:30 p.m. because of the escalating volcanic activity. It was raised further on the same day to Alert Level 3 by 4:00 p.m. and then to Alert Level 4 by 7:30 p.m. Alert Level 5, the highest category on a scale of 0–5, which means that a hazardous eruption is ongoing, was not raised. According to the Philippine Institute of Volcanology and Seismology (PHIVOLCS), the agency mandated to mitigate disasters that may arise from volcanic eruptions, progression from phreatic to a phreatomagmatic eruption took place at about 5:00 p.m., when magma came into contact with groundwater or lake water from the Main Crater Lake (MCL) of Taal Volcano Island (TVI) (PHIVOLCS 2020a). The tall volcanic plume and large associated umbrella cloud of the main eruption resulted in tephra (ash) fall to the north–northeast as far as the capital city of Manila, 65 km from the active vent. By 2:49 a.m. of the following day (13 January 2020), the activity transitioned to lava fountaining. The change in eruptive style was the start of waning activity, characterized by discrete cannon-like explosions that generated 2-km-high bent-over plumes during the rest of the second day and eruption columns of various heights less than 1 km that lasted until 22 January 2020. Steam-laden plumes persisted in the next few weeks with decreasing intensity.

Many local tourists and residents around Taal Lake witnessed the explosive eruption as the event happened on a weekend. As news of the eruption spread, some authors of this paper rushed towards TVI to document the event through photographs and videos, and by sampling tephra for further analysis. The lead authors from the University of the Philippines (UP) have research and public service mandates (Congress of the Philippines 2020). Some are also researchers of the UP Resilience Institute, whose mission is to empower local communities through multidisciplinary actions toward resilience. As such, an independent quick response team was formed to solicit crowdsourced information from the public through social media outlets. Residents affected by the tephra fall, known as ashfall to the layman, were requested through social media to document and collect samples before they were washed out by rain or swept away. In the weeks following the main eruption of Taal Volcano, the team conducted fieldwork to measure tephra fall thickness and collect samples in different locations where tephra falls were reported to have been deposited.

This paper describes the collective work done to document details of the eruption sequence and impacts of tephra fall of the 2020 Taal Volcano eruption. A significant amount of data comprising this paper is owed to the efforts of the general public who immediately reported their observations and contributed tephra samples around Taal Volcano. It was a unique and significant opportunity to engage the public in citizen science, with the crowdsourcing initiative providing the authors with invaluable data while raising awareness through engagement in the observation of volcanic hazards phenomena. As one of the 16 Decade Volcanoes identified by the International Association of Volcanology and Chemistry of the Earth’s Interior (IAVCEI), this work is of significant value, most notably in light of Taal Volcano’s destructive nature and proximity to densely populated areas (Torres et al. 1995), including the country’s National Capital Region, a metropolis inhabited by more than 13 million people (PSA 2021).

## Methodology

To characterize Taal Volcano’s 2020 eruption through its tephra deposits, ground observations, satellite remote sensing, crowdsourcing, field validation, numerical simulations, and laboratory analysis were performed. Each of these methods are described below.

### Ground and satellite observations

Photos taken by residents around Taal Lake, weekend tourists, and passengers of commercial airplanes were reviewed to determine the type of eruption that took place on 12–13 January. Most of these photographs are available from the Internet but some were provided directly to the authors. Time-lapse videos taken by the authors on 13 January from Tagaytay, north of TVI, were also reviewed.

The eruption plume was directly observed and photographed by some of the authors from a distance of about 22 km in the late afternoon of 12 January. The following day, the eruption was observed from the northern ridge of Taal Caldera. Time-lapse videos of the eruption were taken during the observation periods (see Supplementary Files).

Himawari satellite images (Bachmeier 2020) were used to measure the umbrella cloud radius. These were compared with estimates of the eruption cloud height of the cataclysmic event on 12 January determined by the Pacific Tropical Cyclone Center (PTCC) (Perttu et al. 2020; PTCC 2020) and the Cooperative Institute for Meteorological Satellite Studies (CIMSS) (Bachmeier 2020) and used to determine the Volcanic Explosivity Index (VEI) (Constantinescu et al. 2021).

### Crowdsourced accounts and field observations

**Fig. 1:**
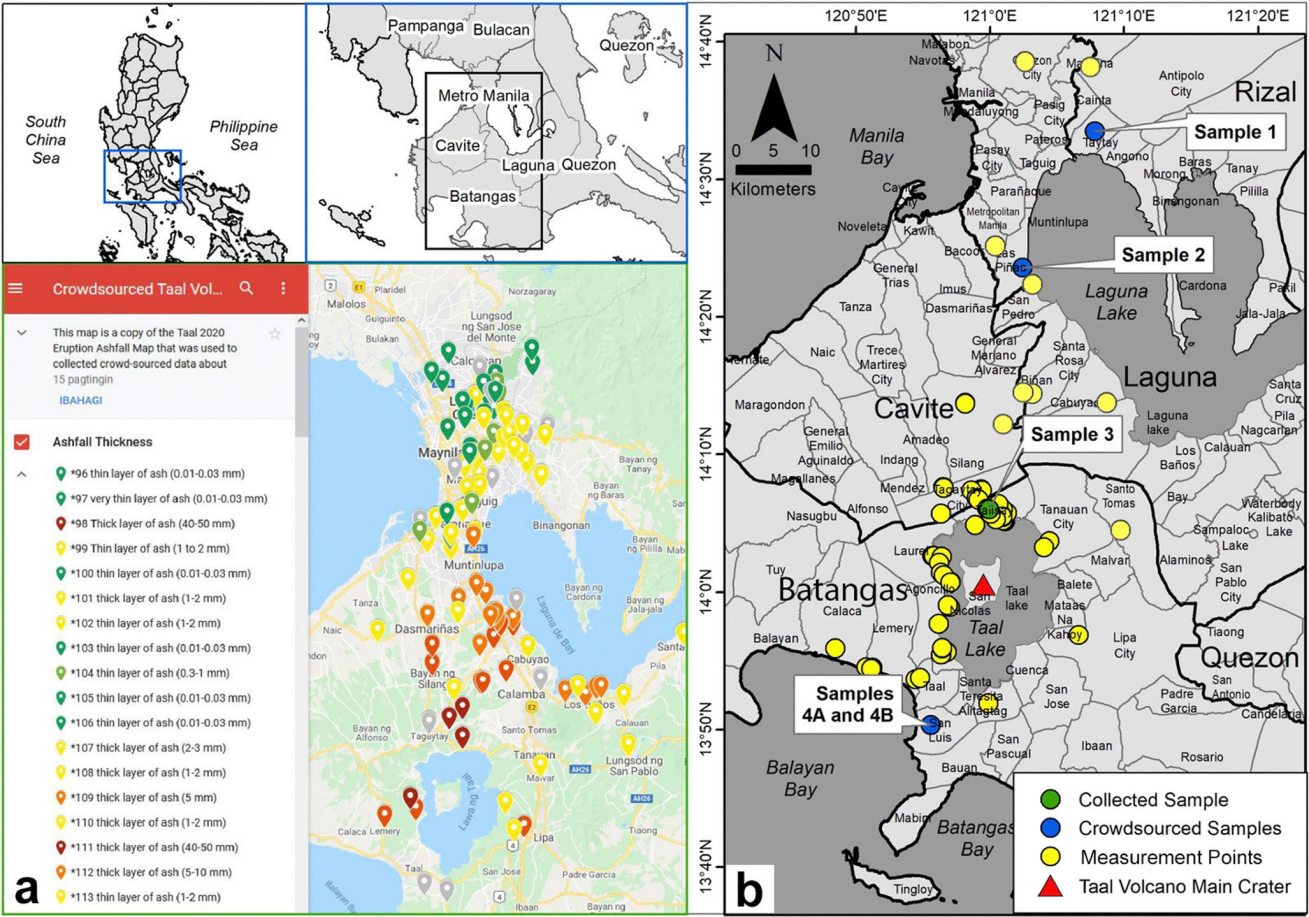
(a) Interactive Google Map showing the locations of ashfall occurrences as reported by netizens. Each pin corresponds to an ashfall thickness in that site: dark green, 0.01 to 0.03 mm; light green, 0.30 to 1.00 mm; yellow, 1.00 to 3.00 mm; orange, 3 to 10 mm; light red, 10 to 30 mm; and dark red, 30–100 mm. No report for ashfall with thickness of 0.03–0.30 mm was received through the Google Map platform. (b) Map showing the location of measurement and sampling points during the field validation

Recognizing that the Philippines remains the leader in social media and Internet usage worldwide (Baclig 2020) and the fact that tephra fall deposits are easily removed by water erosion in tropical regions, an interactive Google Map was created on the morning of 13 January (Fig. 1a) for “netizens,” or citizens who are highly active on social media and other online platforms, to quickly report the pin location and estimated thickness of ashfall deposits in their area. The interactive map also allowed attachment of photos to help visualize the ashfall deposit thickness. Social media posts on Facebook and Twitter containing reports of ashfall were integrated into the Google Map. The advantage of using Google Maps is that contributors could visualize ashfall reports and how their individual pins shaped the data and the map.

The use of hashtags such as #TaalVolcano, #iTalaAngTaalAsh (translation: #RecordTheTaalAsh), and #TaalAshfall were particularly useful in consolidating the posts, tweets, and news regarding the event. Informative discussions on the on-going state of Taal Volcano and its implications were formed in Twitter and Facebook, engaging everyone to participate and spread awareness. In addition, the hashtags were used to pinpoint the location of severely affected areas needing donations, relief efforts, and rescue.

The authors also focused on public communication, leading forums, and producing infographics about the eruption. The discussions on the original posts in social media lasted for over a week on Twitter and Facebook. The calls for crowdsourced data were retweeted by a media outfit, a science organization (AGHAM, Advocates of Science and Technology for the People) as well as local and international scientists with one post garnering a total of 372,003 impressions, 86,206 engagements, 1954 likes, and 1436 retweets. The interactive Google Map reached over 3000 views and received 247 unique submissions.

All reports were vetted to assess the quality of thickness measurements and only those with associated photographs were considered. Of the 247 reports, 167 thickness measurements were deemed accurate. Contour lines were drawn on points of similar tephra fall deposit thicknesses to construct a GIS isopach map.

A series of field surveys were conducted by the Quick Response Team of the UP National Institute of Geological Sciences and UP Resilience Institute in the following municipalities: (1) Agoncillo, Batangas; (2) Calaca, Batangas; (3) Laurel, Batangas; (4) Lemery, Batangas; (5) San Nicolas, Batangas; (6) Taal, Batangas; (7) Talisay, Batangas; (8) Tanauan, Batangas; (9) Silang, Cavite; and (10) Tagaytay City, Cavite. Observation and measurement points were chosen to show optimal preservation of the deposits (Fig. 1b). Tephra fall thicknesses were most reliable when measured on house and car rooftops, bridge columns and railings, road and fence ledges, fallen trees, and tombs in cemeteries. The total lockdown in some places due to the eruption significantly reduced anthropogenic activity, reducing reworking of the tephra and preserved the tephra fall deposits in many areas for weeks. Tephra samples were collected and stored in resealable plastic bags with several samples solicited from colleagues residing in towns where tephra fall occurred.

### Eruption parameter calculations

*TephraFits* was used to model the Taal Volcano’s 2020 main eruptive episode based on the crowdsourced and surveyed tephra fall data. It is a Matlab function, a collection of codes dedicated to the characterization of tephra fall deposits such as *AshCalc* (Daggitt et al. 2014) and *TError* (Biass et al. 2014). The *TephraFits* program enables the user to quickly compute eruption parameters including the deposit volume and mass, VEI, magnitude, and rates of thickness-decay away from the source and allows for better eruption classification using deposit-based schemes (Biass et al. 2019). Outputs generated by the *TephraFits* algorithm follow three well-known and frequently used fitting methods, which are the exponential, power-law, and Weibull integration models.

#### Exponential model

The exponential model is an integration strategy that describes the relationship between the deposit thickness, *T*, as a function of the root isopach area, *x* as follows (Eq. 1):

1 $$ T(x)=ce^{-mx}  $$

where *c* represents the theoretical maximum thickness located at the vent and *m* describes the rate of decrease in tephra deposit thickness (Pyle 1989; Biass et al. 2019). This equation is linearized by taking the logarithm of both sides of the equation and least squares regression is applied to get the values for parameters, *c* and *m* (Daggitt et al. 2014).

Multiple exponential segments are applied to the data so that the exponential law allows for modelling deposits of varying thinning rates with distance away from the vent as displayed by many well-constrained tephra fall deposits (Pyle 1989; 1990; Fierstein and Nathenson 1992; Bonadonna and Houghton 2005; Watt et al. 2009). This integration approach is effective for simple approximations such as determining the minimum volume of a deposit given sparse data points (e.g., Pyle 1995; 1999; Legros 2000; Sulpizio 2005) (Daggitt et al. 2014).

#### Power-law model

The power-law model is another fitting method that describes the relation between thickness, *T*, as a function of root isopach area, *x*, as follows (Eq. 2):

2 $$ T(x)=cx^{-m}  $$

where *c* represents a linear scaling factor and *m*, characterizes the rate of decrease in tephra deposit thickness (Bonadonna et al. 1998; Bonadonna and Houghton 2005). Similar to the exponential model, this equation is linearized by taking the logarithm of both sides of the equation and least squares regression is applied to get the values for parameters, *c* and *m*. The main disadvantage of using the power-law model is that *T(x)* is not integrable between 0 and $\infty $. Thus, the proximal and distal limits of integration have to be selected (e.g., Bonadonna and Costa 2012) (Daggitt et al. 2014).

#### Weibull model

Of the three fitting methods, the Weibull model was proposed more recently as it combines the advantage of the exponential model being integrable between 0 and $\infty $ and the power-law model’s use of variable rates of decrease in deposit thickness (Bonadonna and Costa 2012). The model describes the relationship between thickness, *T*, and root isopach area, *x*, as follows (Eq. 2):

3 $$ T(x)=\theta(\frac{x}{\lambda})^{k-2}e^{(\frac{x}{\lambda})^{k}}  $$

The additional parameter, *λ*, allows the Weibull model to capture variation in the deposit’s thinning rate, which in the exponential model necessitates multiple segments (Daggitt et al. 2014).

For the exponential, power-law, and Weibull fitting, the implementation strategies recommended by Biass et al. (2019) were used in this study. A 10% error was applied to all thickness and diameter measurements and a 20% error on the distal limit (*C*). Several iterative runs were conducted while observing strict statistical measures and ensuring critical interpretation that would match geologically realistic values. For example, the *𝜃* output for the Weibull Fit was ensured to agree with the thickness ($\sim $ 1 m) and maximum grain size values ($\sim $ 11 cm) of tephra fall deposits observed near the crater rim where there were no identified base surge deposits (Lagmay et al. 2021). Values were compared to tephra fall deposits from other volcanoes in the global dataset to ensure they were realistic (Bonadonna and Costa 2013; Daggitt et al. 2014; Biass et al. 2019). Further sensitivity analyses were performed for output values, especially for the distal limits where thickness values are harder to constrain (sub mm) and preservation is short-lived. A list of the final selected input parameters used for the results presented in this work is shown in Table 1.

**Table 1:** List of input parameters used to calculate volume, VEI, eruption height, and eruption magnitude based on tephra fall thickness and grain size parameters of tephra fall samples

	Isopach			Isopleth		
	Exponential	Power-law	Weibull	Exponential	Power-law	Weibull
Isopachs used (cm)	45, 10, 1.5, 0.5, 0.3, 0.10, 0.05			—		
Isopleths used (cm)	—			1.65, 0.325, 0.13, 0.075		
Break in slope (BIS)	4	—	—	2	—	—
Distal limit (*C*)	—	130	—	—	—	—
*λ* range	—	—	5–20	—	—	2–10
*n* range	—	—	0.2–20	—	—	0.5–2

A 10% error was applied to all thickness and diameter measurements and a 20% error on distal limit (*C*). Values of *λ* and *n* are within the ranges recommended by Bonadonna and Costa (2013)

Furthermore, this study used the Weibull method to estimate the plume height according to equation 7 of Bonadonna and Costa (2013). The modeled eruption height values were then compared with those measured from satellite data (Perttu et al. 2020; Bachmeier 2020). Magnitude-related parameters such as thickness half-distance, *b*_*t*_ and *λ*_*TH*_, as well as intensity-related parameters, in particular thickness half-clast, *b*_*c*_ and *λ*_*ML*_ (Biass et al. 2014), were used to classify the 2020 Taal eruption according to the schemes of Pyle (1989) and Bonadonna and Costa (2013).

#### Limitations

Circumspection on the use of *TephraFits* is hereby noted. Our calculations are based on crowdsourced and field survey data, which are relatively scarce point observations of the entire tephra fall deposit. More thickness and maximum grain-size measurements could have been added, but lockdown restrictions due to the raised alert level for Taal Volcano and later for COVID-19 prevented further collection of data. Most of the crowdsourced data were visually estimated from the photos submitted whereas field surveys were delayed by 1–2 weeks. Erosion by wind and surface water runoff may have reduced the thickness of the fall deposits. Furthermore, the data points were subjectively contoured to generate isopach and isopleth maps used to constrain transport and depositional processes. As such, many sources of uncertainties interacted and propagated to the final results presented in this work (Biass et al. 2019). Nonetheless, these caveats guided us in the critical use and interpretation of the models, ensuring that they reflect geological reality and direct observations of Taal Volcano’s main eruption.

### Duration of peak activity

**Fig. 2:**
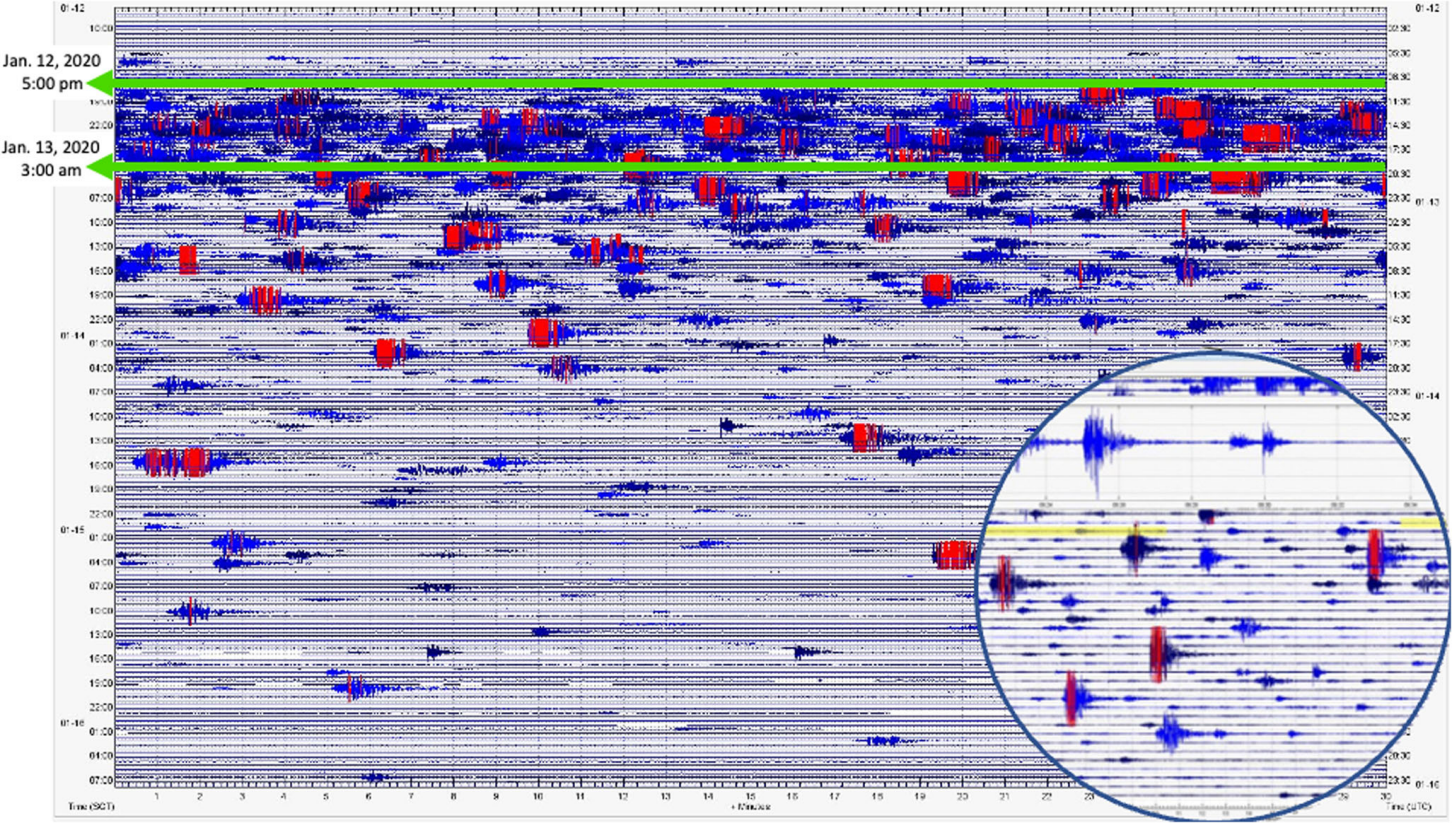
Seismogram from a public seismic network (PSN) station in Dasmarinas, Cavite, available from FDSN. Blue lines are seismic waveforms whereas the red lines are clipped waveforms due to their high amplitude. The estimated period of intense seismic activity corresponding to the development of the 17–21-km-high plume is approximately from 5 p.m. of 12 January to 3 a.m. the following day. The inset circle is the wave panel showing a magnified view of the recorded waveform with amplitude represented in counts

The duration of peak activity was determined from the review of proximal seismic data recorded by a station north of TVI in Dasmarinas, Cavite. Data were retrieved from the International Federation of Digital Seismograph Networks (FDSN) (Fig. 2), which provides open access to seismic data from the Philippines. The seismic records came from the nearest station to Taal, which is part of a growing network of low-cost but professional seismometers (Anthony et al. 2018; Bent et al. 2018; Holmgren and Werner 2021; Subedi et al. 2020) in the country, owned and operated by private entities and citizen scientists (Aurelio et al. 2020a; 2020b). The estimate of the duration of peak activity was compared with the accounts of eruptive activity in official bulletins of PHIVOLCS (Table 2), reports from the Global Volcanism Program, news articles, social media posts, and airlines and ground observations by the authors.

**Table 2:** Timeline of events up to 22 January 2020 according to PHIVOLCS bulletins and cross-referenced with other sources

Date	Time	Alert level	Event description	Ashfall areas
12 Jan 2020	1:00p.m.	1	Increased steaming activities observed in five places in the main crater with the most active part exhibiting a phreatic explosion with a 100-m-high plume	
	2:00p.m.	1	Booming sounds were heard in Talisay, Batangas	
	2:04p.m.	1	Increase in activity within the MCL and produced a 1-km-high eruption column	Ashfall observed in the southwestern portion of TVI
	2:30p.m.	2	Alert level to 2	
	4:00p.m.	3	Alert level to 3	
	5:30p.m.	3	Activity intensified with sustained eruption generating a steam-laden tephra column 10–15 km high	Wet ashfall fell to the north and reaching as far as Quezon City
	7:30p.m.	4	Alert level to 4	Ashfall was observed in several places
13 Jan 2020	2:49a.m.	4	Magmatic eruption with lava fountaining observed	
	3:20a.m.	4	PHIVOLCS bulletin update of areas affected by ashfall	
	4:28a.m.	4	Lava fountaining ceased. Eruption quickly resumed with weak sporadic fountaining and hydrovolcanic activity within the main crater persisted with 2-km-high steam-laden plume	
	4:00p.m.	4	Newly formed lateral vents were discovered in the northern flank of the main crater island where 500-m lava fountains emanated	Heavy ashfall to the southwest, experienced mainly in Cuenca, Lemery, and Taal, Batangas
14 Jan 2020	8:00a.m.	4	Activity within the last 24 h has been defined by sustained eruption in the main crater due to hydrovolcanic and magmatic activity	Heavy ashfall from the ongoing eruption has fallen in Lemery, Talisay, Taal, and Cuenca, Batangas
	1:00p.m.	4	Activity produced a 800-m-high dark gray steam-laden plume	Plume drifted southwest from the main crater
15 Jan 2020	8:00a.m.	4	The sustained eruption produced a 1-km-high dark gray steam-laden plum.	Ash dispersed southwest of the main crater
	5:00p.m.	4	The continuous eruption produced a 700-m-high dark gray steam-laden plume. “Drying-up” of portions of the Pansipit River has been reported. Acquired satellite images also show water from the MCL has disappeared and new vent craters have developed in the north flank of the volcano	Plumes drifted southwest from the main crater
16 Jan 2020	6:17a.m.	4	Eruptive activity produced a 500-m-high dark gray ash plume	Ash dispersed west–southwest from the main crater
	6:21a.m.	4	Eruptive activity produced a 800-m-high dark gray ash plume	Ash dispersed west–southwest from the main crater
	8:00a.m.	4	Activity within the last 24 h has diminished to weak emissions of steam-laden plumes 700m high	Ash dispersed southwest from the main crater
	5:00p.m.	4	Activity within the last 8 h has been defined by weak emission of steam-laden plumes 800m high. Nine distinct weak explosions were recorded	Plumes drifted southwest from the main crater
17 Jan 2020	8:00a.m.	4	Activity within the last 24 h was defined by consistent steam emissions and irregular explosions that produced dark gray ash plumes 100–800m high	Ash dispersed west–southwest from the main crater
	5:00p.m.	4	Activity since 8:00a.m. was distinguished by weak emissions of steam-laden plumes 800m high. Five distinct weak explosions were recorded	Plume drifted southwest from the main crater
18 Jan 2020	8:00a.m.	4	Activity within the last 24 h was defined by consistent steam emissions and irregular explosions that produced white to dirty white ash plumes 50–600m high	Ash dispersed southwest from the main crater
	6:00p.m.	4	Activity since 8:00a.m. was distinguished by a weak emission of steam-laden plumes 500–800m high. Two distinct weak explosions were recorded	Plume drifted southwest from the main crater
19 Jan 2020	8:00a.m.	4	Activity within the last 24 h was defined by consistent steam emissions and irregular explosions that produced white to dirty white ash plumes 500–1000m	Ash dispersed southwest from the main crater
	6:00p.m.	4	Activity since 8:00a.m. was distinguished by a weak emission of steam-laden plumes 300–500m high	Plume drifted southwest from the main crater
20 Jan 2020	8:00a.m.	4	Activity within the last 24 h was defined by consistent steam emissions and irregular explosions that produced ash plumes 500–1000m high	Ash dispersed southwest from the main crater
21 Jan 2020	8:00a.m.	4	Activity within the last 24 h was defined by weak emissions of white steam-laden plumes 500–600m high	Ash dispersed southwest from the main crater
22 Jan 2020	8:00a.m.	4	Activity within the last 24 h was defined by weak emissions of white steam-laden plumes 50–500m high	Ash dispersed southwest from the main crater
	4:00p.m.	4	Since 5:00a.m., no ash emissions were observed based on visual observations and seismic records. However, unconsolidated ash in Taal was remobilized by strong-low level winds, and based on reports by multiples airlines, reached approximately 5800m.	Remobilized unconsolidated ash drifted southwest affecting Lemery and Agoncillo

Elevations are above mean sea level. *Abridged

### Laboratory analysis

Samples used for grain-size analysis conducted at the UP National Institute of Geological Sciences (UP NIGS) came from those collected during fieldwork and those solicited from colleagues (see Fig. 1b for locations). For grain size analysis, the samples were oven dried for 6–8 h and reduced to 100 g using coning and quartering to eliminate systematic biases. The maximum grain sizes of predominantly equant tephra grains utilized in the isopleth maps came from twenty samples that were handpicked and measured using a micrometer. Manual sieving was then carried out on three samples using the No. 18 (1.00 mm, 0*ϕ*), No. 35 (0.50 mm, 1*ϕ*), No. 60 (0.25 mm, 2*ϕ*), No. 120 (0.125 mm, 3*ϕ*), and No. 230 (0.063 mm, 4*ϕ*) test sieves of the U.S.A. Standard Test Sieves ASTM E-11-20 (International ASTM 2014) to determine the size frequency distribution. Using an analytical balance, size fractions were weighed, and those data were plotted on a standard grain-size distribution graph. The manually sieved tephra samples were then sonicated to separate aggregates for componentry analysis using a Zeiss stereomicroscope. The relative abundance of each component was assessed by grain counting at least 300 particles per size fraction (> 125–250 μm, 250–500 μm, 500 μm–1 mm, 1–2 mm, 2–4 mm, and > 4 mm) for each sample.

Two additional samples were sent to the Laboratoire Magmas et Volcans, University Clermont Auvergne (LMV-UCA), for further analyses on size frequency distribution, texture, morphology, and geochemistry. Both samples were collected at the same location 20 km from the eruptive vent (sample 4A and sample 4B) on 13 January (see Fig. 1b for location). The first (sample 4A) was collected at 10:22 a.m. and the second (sample 4B) at 1:05 p.m. Grain sizes for both samples were first measured by manual sieving following the same procedure as described above. Particle size distribution (PSD) was measured on size fractions below 1 mm in diameter using a MALVERN Mastersizer 300 laser diffractometer, following the Thivet et al. (2020) procedure.

The following textural and chemical analyses were then performed on the sample grain-size modes (125–90 μm). Componentry was determined on the 2 samples from epoxy-impregnated polished sections using a JEOL JSM-5910 LV scanning electron microscope (SEM). The 2D internal textures of the particles were investigated with backscattered electron (BSE) imagery with an acceleration voltage of 15 kV. At least 100 particles were counted for each sample. The 3D particle surfaces were imaged using a CARL ZEISS Supra 55/55 VP field emission (FE) SEM and using secondary electron (SE) imagery, with an acceleration voltage of 3 kV.

Finally, 2D ash morphology was quantified using a MALVERN Morphologi G3 morpho-grainsizer following the Thivet et al. (2020) procedure. Measurements were performed on grain size modes of both samples. At least 1000 particles were analyzed for each sample and particle shapes were automatically measured by the instrument. The different shape parameters used in our study are described in Thivet et al. (2020). In our study, we represented particle morphology through two distinct roughness parameters, solidity (SLD) and convexity (CVX), which represent the morphological (particle scale) and the textural scale (smaller scale) roughness of the particles, respectively. A perfectly round or square particle has a SLD and CVX value of 1. On the other hand, SLD and CVX decrease as soon as shape irregularities appear.

In situ glass compositions were determined from microlite-free juvenile particles, using a Cameca SxFiveTactis electron probe micro-analyzer (EPMA), with an acceleration voltage of 15 kV, current intensity of 8 nA, and a laser beam of 10 microns, following the Gurioli et al. (2018) procedure.

## Results

### Crowdsourced accounts

The majority of crowdsourced reports came from Metro Manila with fewer netizen reports received from the CALABARZON (Cavite, Laguna, Batangas, Rizal, and Quezon) Region. This can be attributed to the difference in population density between the National Capital Region and these adjacent provinces, and that most of CALABARZON was heavily affected by the eruption (Fig. 1). All residents within 14 km of TVI were ordered to evacuate and many other residents outside of this area voluntarily relocated to safety. Partial power interruptions in the municipalities of Cavite, Laguna, and Batangas lasting for days after the eruption (Del Castillo et al. 2020) likely contributed to fewer ashfall reports from these locations. Despite the smaller number of data reported from proximal locations, we consider the dataset robust enough when augmented with additional field observations.

The thinnest tephra fall deposits were reported north of Metropolitan Manila as well as in the provinces of Rizal, Bulacan, and Pampanga. These deposits were observed as dispersed tephra particles with a thickness of < 1 mm that were hardly visually discernible, particularly in urban to suburban areas.

**Fig. 3:**
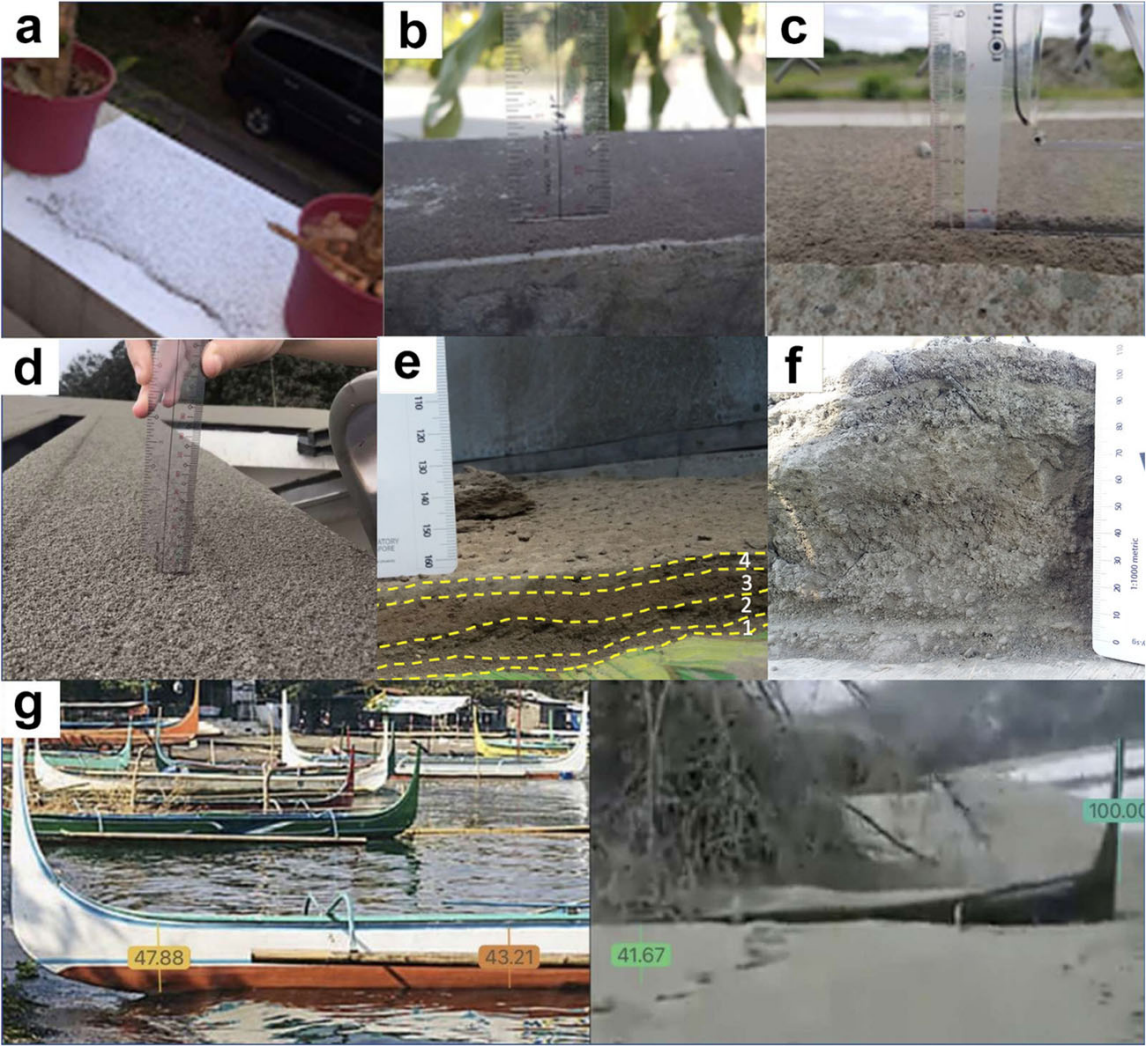
Representative photos of tephra fall deposits with varying thicknesses crowdsourced from netizens and from our fieldwork: (a) < 1 mm in Quezon City; (b) 1 mm in Quezon City; (c) 2.5 mm in Lipa City, Batangas; (d) 5 mm at Westgrove Heights, Silang, Cavite; (e) 40 mm in Talisay, Batangas (dashed lines represent different layers in the deposit); and (f) 110 mm in Agoncillo, Batangas; (g) 400–500 mm at the north shore of TVI. The two photos are of a banca, a small boat, typically used in the island. One photo was taken before the eruption; the other is a video screenshot from Facebook posted by GO Batangas on January 13. Numerical values in the photos refer to heights (in centimeters)

Thicker deposits, with thickness of ≥ 1 mm, were more apparent and easily documented by netizens. These could be measured with a standard ruler or better estimated in photographs, and were reported in southern Metropolitan Manila and some portions of the provinces of Rizal, Cavite, and Laguna. Tephra fall deposits progressively thicken towards the eruption source. Deposits with thicknesses of at least 1 up to 10 mm (Fig. 3c, d) fully blanketed the surface of roads, roofs, car hoods, and leaves of plants.

Tephra fall deposits closer to the source in Cavite, Laguna, and Batangas were much thicker with thicknesses of 10 to 30 mm (Fig. 3e, f). Objects and structures situated outdoors were heavily blanketed with tephra. Public roads were also fully covered with thick tephra that required clearing for safe use. Netizens from these areas reported “smoking” ash outside of their homes as wind remobilized and dispersed tephra particles.

The thickest tephra fall deposits were reported in the municipalities and cities of Batangas immediately surrounding Taal Lake. These include Agoncillo, Calaca, Laurel, Lemery, Lipa, San Nicolas, Talisay, Taal, and Tanauan. Deposits 40 to 50 mm thick heavily damaged vegetation (Fig. 3). No netizen reports of ashfall from TVI were received.

### Field observations

On 12 January, tephra from the eruption reaching plume heights of 17–21 km was first deposited southwest of Taal Volcano (Lagmay et al. 2021). A few hours later, the plume shifted north–northeastwards and wet tephra fall showered cities and municipalities 70 km north–northeast of the main crater. On 13 January, continuous activity at the volcano generated plume heights up to 2 km above sea level and an eruption cloud that drifted to the southwest of the volcano.

The tephra fall deposits exhibited parallel layerings made distinct by changes in color and grain size. In proximal areas, layers with ash-sized (< 2 mm in diameter) to lapilli-sized (> 2 mm diameter) lithic, vitric, and crystal fragments are observed in the lower layers. North of the volcano island, in Silang, Tagaytay, Talisay, and Tanauan, deposits have thicknesses from 1 to 4 cm with at least four layers. In Talisay, Batangas, 9 km from the crater, a deposit was observed to exhibit a light gray bottom layer (layer 1) overlain by a dark gray ash deposit (layer 2) with coarse accidental lithic lapilli, vitric grains, and free crystals (Fig. 3e). The third layer (layer 3) is made up of brown ash and is the thickest. Finally, light gray ash makes up the topmost layer (layer 4). In Tagaytay, a similar deposit was observed but with the bottom-most light gray layer absent. Furthermore, most of the observed proximal deposits contained accretionary lapilli.

### Deposit volume calculation

**Fig. 4:**
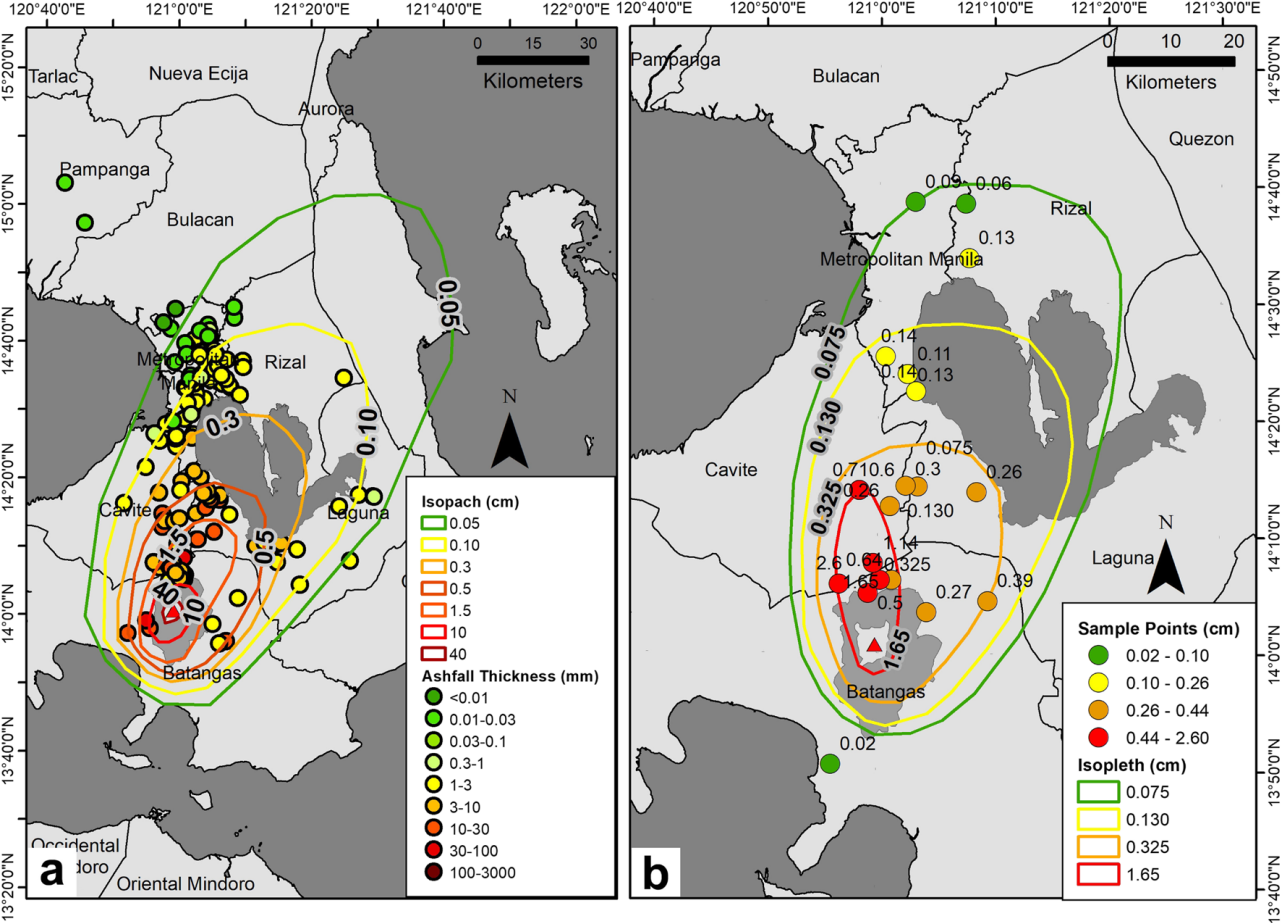
Isopach (a) and isopleth (b) maps generated from vetted crowdsourced accounts and field data corresponding to the 12 January to early morning (about 4:00 a.m.) of 13 January eruption of Taal Volcano

Thickness reports and field data reveal the approximate distribution of the tephra fall deposits of the 12–13 January eruption of Taal Volcano (Fig. 4a). Ellipticity for all isopach areas range from 0.15 to 0.56, which implies elongated tephra fall deposition rather than an axial symmetry. The ellipticity of the tephra deposit is attributed to winds blowing toward the north–northeast at higher levels in the atmosphere during the main part of the eruption (i.e., 1:00 p.m. 12 January to 4:28 a.m. 13 January).

A total isopach map area of 8605 km^2^ was covered by tephra fall after the 12 January eruption based on 0.05-cm depth, crowdsourced data, and field observation. Within this area, seven isopach contours were drawn (Fig. 4a) corresponding to the reported thickness ranges of tephra fall after the eruption.

The isopleth contours were delineated based on the maximum grain sizes measured from the samples (Fig. 4b) gathered during field validation. A gradual decrease of tephra grain sizes from the TVI to the surrounding communities can be observed on the map, with the smallest (0.02–0.10 cm) grain sizes sampled just within Metro Manila. The trend of decreasing tephra grain size also followed the north–northeast wind direction at the time.

The thickness and maximum grain size values used for the isopach and isopleth contours, respectively, are listed in Table 3 with their corresponding area in square kilometers. The thickest isopach contour, 40 mm, approximately encloses TVI and has a distance, *r*, of approximately 2 km away from the main crater. At this distance, the thickness of the tephra deposit was determined from the nearly complete burial of the hull of a banca, a traditional boat used in Taal Lake (Fig. 3g). The thinnest measurement is ≤ 1 mm, reported by netizens in Quezon City in Metro Manila and in the Rizal/Quezon Province boundary.

**Table 3:** Thickness values of isopach contours and maximum grain size values of isopleth contours

Isopach	Maroon	Red	Red orange	Orange	Yellow	Light Green	Green
Thickness (cm)	45	10	1.50	0.50	0.30	0.10	0.05
Area (km^2^)	22.90	123.24	436.77	764.24	1107.91	2285.22	3864.89
Isopleth	Red		Orange		Yellow		Green
Max grain size (cm)	1.65		0.325		0.13		0.075
Area (km^2^)	216.57		699.98		1092.35		1307.47

The corresponding areas covered by the isopach and isopleth contours are correspondingly listed

**Fig. 5:**
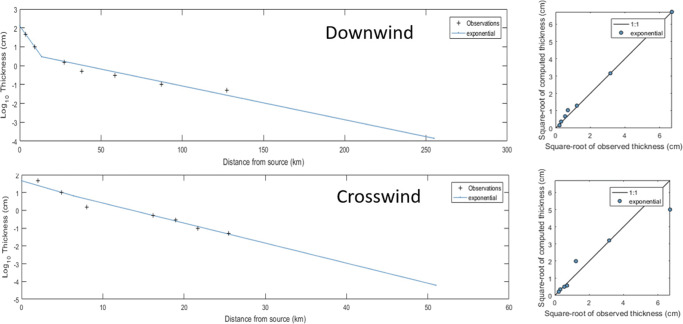
Thickness half-distance exponential plot of the downwind and crosswind segments for Taal Volcano’s tephra fall deposits

The isopach contours show exponential thinning of tephra downwind with a 1.40-km thickness half-distance for the proximal segment and 9.49-km half-distance for the distal segment. Along the crosswind direction, the tephra fall deposit also thins exponentially with a 1.28-km thickness half-distance for the proximal segment and 1.50-km half-distance for the distal segment (Fig. 5).

**Fig. 6:**
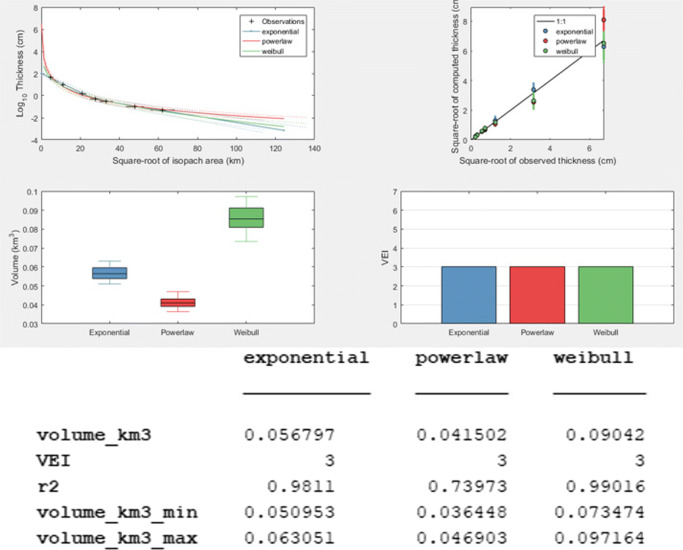
Results of the *TephraFits* model calculations using the exponential, power-law, and Weibull methods

Results on the volume of Taal Volcano’s 2020 tephra fall deposit differ based on the model used (Fig. 6). The exponential model yields a volume of 0.057 km^3^, whereas the power-law model calculates a value of 0.042 km^3^. The Weibull model gives the largest volume of 0.090 km^3^. All are within the volume range of 0.01–0.1 km^3^, which translates to a VEI of 3.

Using the probabilistic approach with 90% confidence intervals or the 5–95th percentile intervals, the range of volumes become 0.051–0.063 km^3^ (exponential), 0.036–0.047 km^3^ (power-law), and 0.073–0.097 km^3^ (Weibull), which shows that the Weibull fit is sensitive to uncertainties regarding isopach thickness and the square root of the isopach area. In the Weibull fit, the 95th percentile of the distribution results in a volume of 0.097 km^3^, which gets very close to the volume that corresponds to VEI 4 (0.1–1 km^3^).

**Fig. 7:**
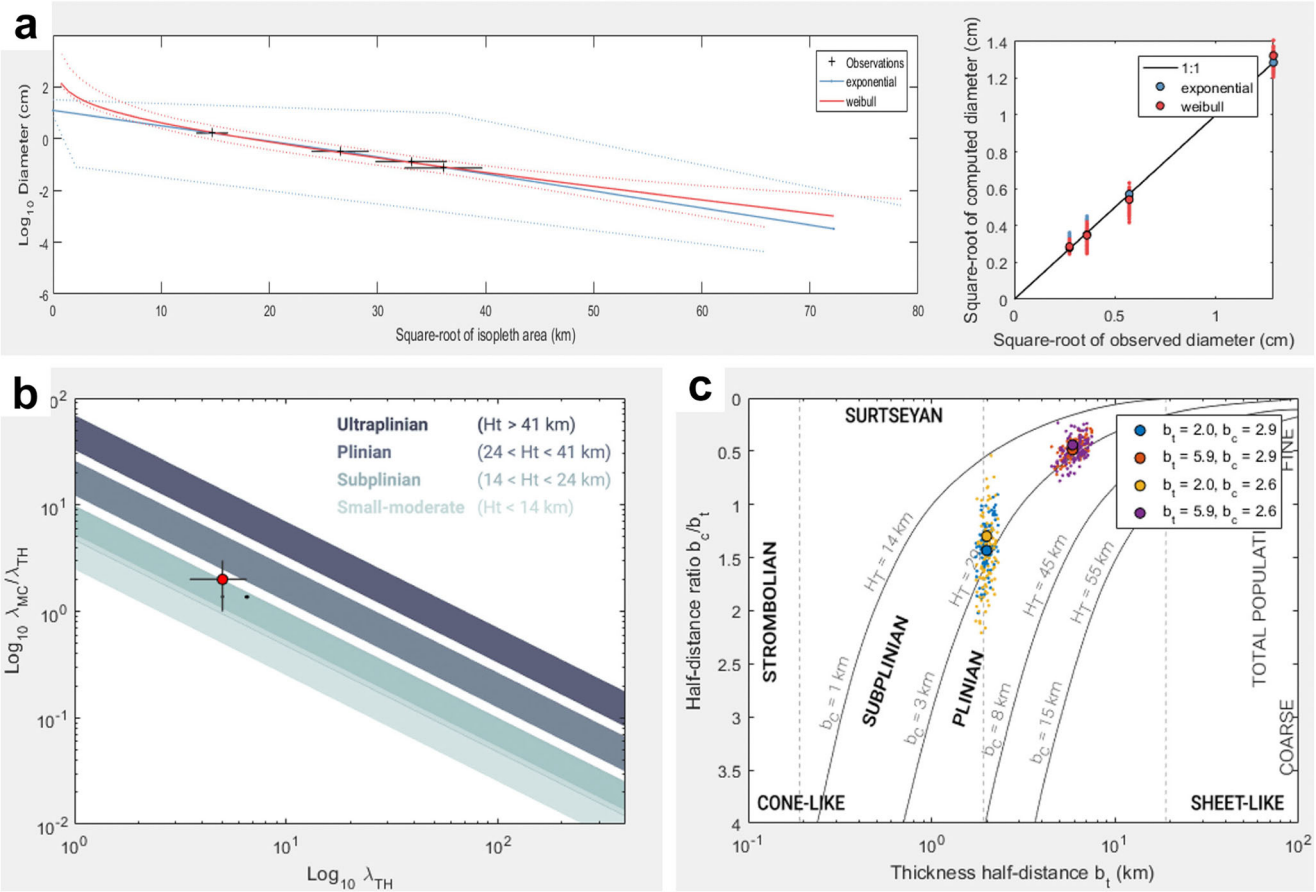
Results of the *TephraFits* calculations for isopleth data (a) and the classification schemes according to Bonadonna and Costa (2013) (b) and Pyle (1989) (c). Both plots in the sub-plinian field

*TephraFits* calculations using the Weibull method based on isopleth data show a median eruption height of 17.8 km above vent. Considering typical uncertainties of 20*%* for determining plume heights (Bonadonna and Costa 2013), the range of plume heights determined for the main eruption of Taal Volcano is 14.2–21.3 km above vent. In terms of the classification of the eruption, we calculate *λ*_*TH*_ and $\frac {\lambda _{MC}}{\lambda _{TH}}$, as well as thickness half-distance *b*_*t*_ and thickness half-distance over thickness half-clast ($\frac {b_{t}}{b_{c}}$) values that plot in the sub-plinian fields of Pyle (1989) and Bonadonna and Costa (2013) (Fig. 7).

### Tephra sample characterization

**Fig. 8:**
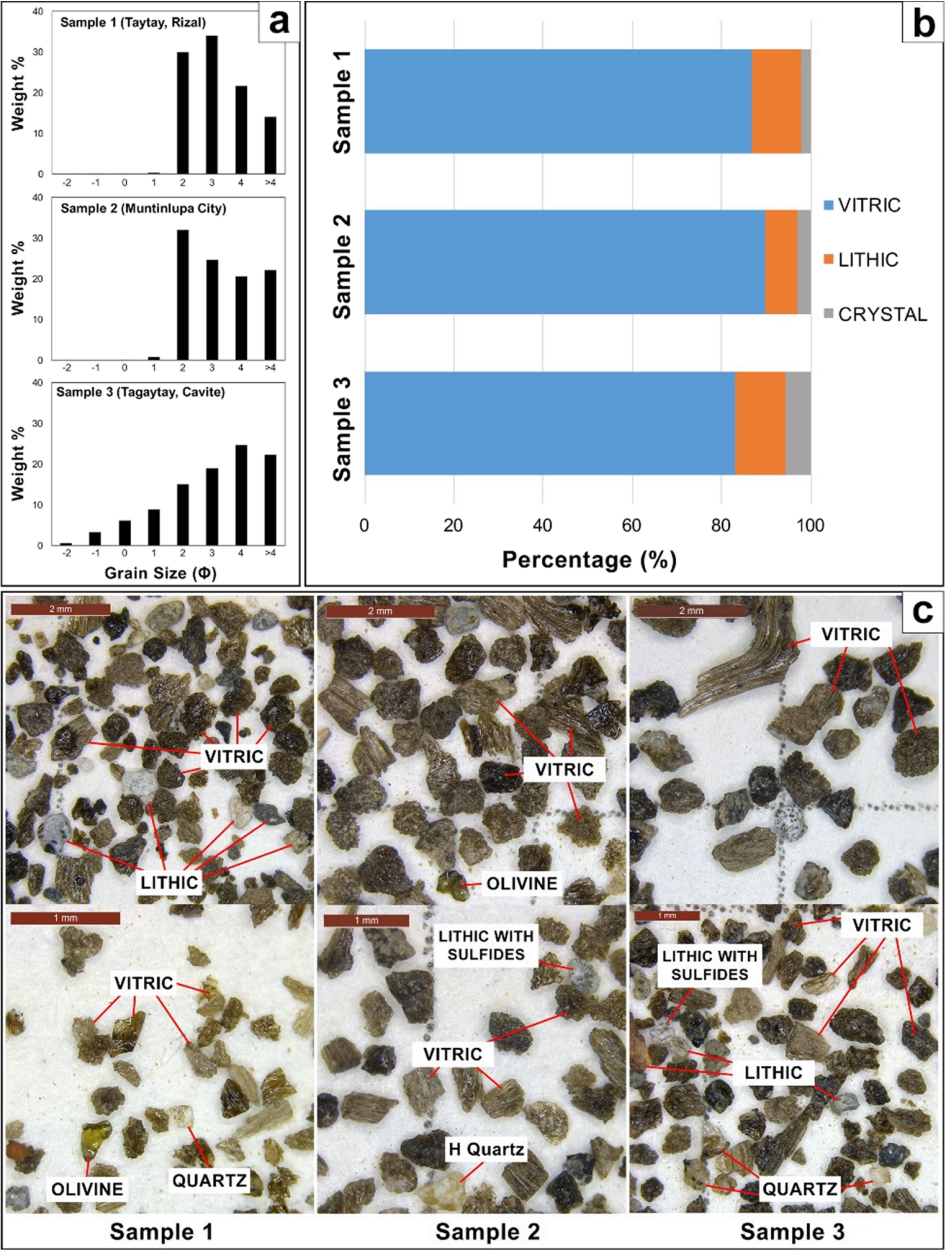
Samples 1, 2, and 3 analyzed at UP-NIGS. (a) Grain-size distribution for the tephra fall deposit in different localities. (b) Relative percentage of the tephra components. (c) Tephra components including vitric, lithic, and crystals (olivine, quartz), as well as secondary quartz (H quartz) and sulfides adhering to the rock fragments

Grain-size distributions of the tephra fall deposits from samples collected north–northeast of Taal Volcano show a unimodal distribution (Fig. 8a). The grain-size frequency plot for the most proximal deposits shows an asymmetrical distribution, skewed towards the finer grain sizes. Coarser tephra grains were absent for the two distal deposits collected in Muntinlupa, Metro Manila, and Taytay, Rizal, with grain-size frequency plots also displaying a single mode at 125–500 μm.

**Fig. 9:**
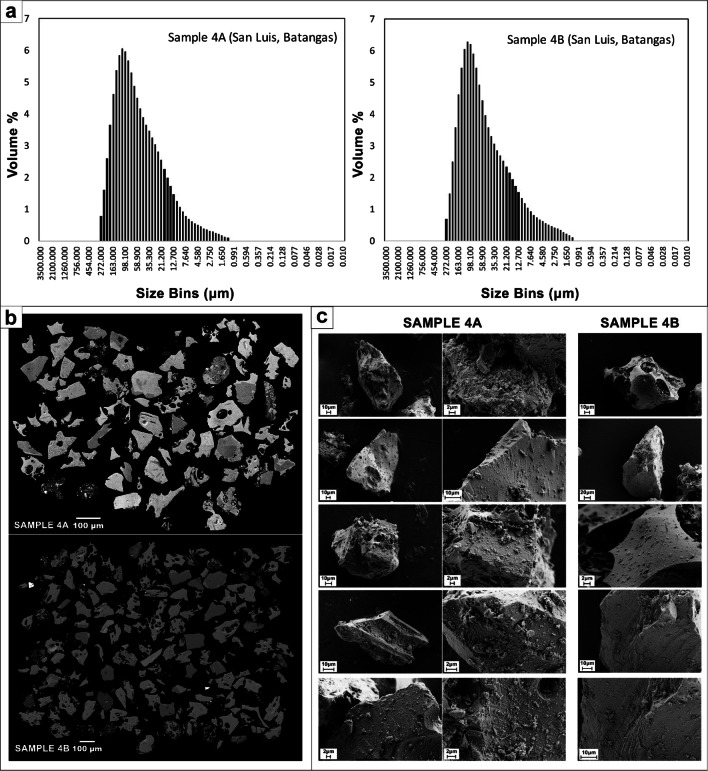
Samples 4A and 4B analyzed at LMV-UCA. (a) Grain-size distribution from laser diffraction measurements, (b) BSE images, (c) SE images

Grain-size distributions of the samples collected south–southwest of Taal Volcano (Fig. 9a) range between 300 μm (1.75 *ϕ*) and 1 μm (10 *ϕ*) and show a single mode at 125–190 μm (3–3.5 *ϕ*). These volume distributions (which reflect mass distributions assuming a constant particle density for each grain size bin) are asymmetric and skewed toward relatively coarse grain sizes. Note that both bulk samples contained aggregates that were crushed during the analysis. These aggregates were irregular in shape with no specific internal grain-size organization.

Binocular microscope analysis showed that the 2020 Taal Volcano tephra consists of volcanic glass (vitric), accidental lithic fragments, crystals, and crystal fragments (Fig. 8c). Grain counting reveals that volcanic glass is the dominant component in all the samples. Volcanic glass or the vitric components consist of about 83–90%. Lithic grains only represent 7–11%, whereas crystal fragments compose less than 6% of the tephra material (Fig. 8b).

Vitric components are translucent light brown to black. The grains vary from blocky to fluted to scoriaceous forms. Lithic fragments, on the other hand, include whitish yellow hydrothermally altered fragments, sub-rounded volcanic rock fragments, and reddish-orange oxidized grains. Crystal fragments of olivine, quartz, and plagioclase were observed as free crystals embedded within glass. Sulfides were also observed in some of the hydrothermal fragments (Fig. 8c).

Backscatter electron (BSE) image analysis of each of the grain size modes of samples 4A and 4B (Fig. 9b) was used to identify different components, including juvenile glassy (sideromelane) particles (80%), juvenile microlite-rich (tachylite) particles (10%), scarce phenocrysts of olivine and plagioclase (10%), and very scarce non-juvenile hydrothermal fragments (< 1%).

Scanning electron (SE) images (Fig. 9c) confirmed the presence of abundant fine adhering particles. The surfaces of juvenile glassy particles are typically composed of stepped features and microfractures.

**Fig. 10:**
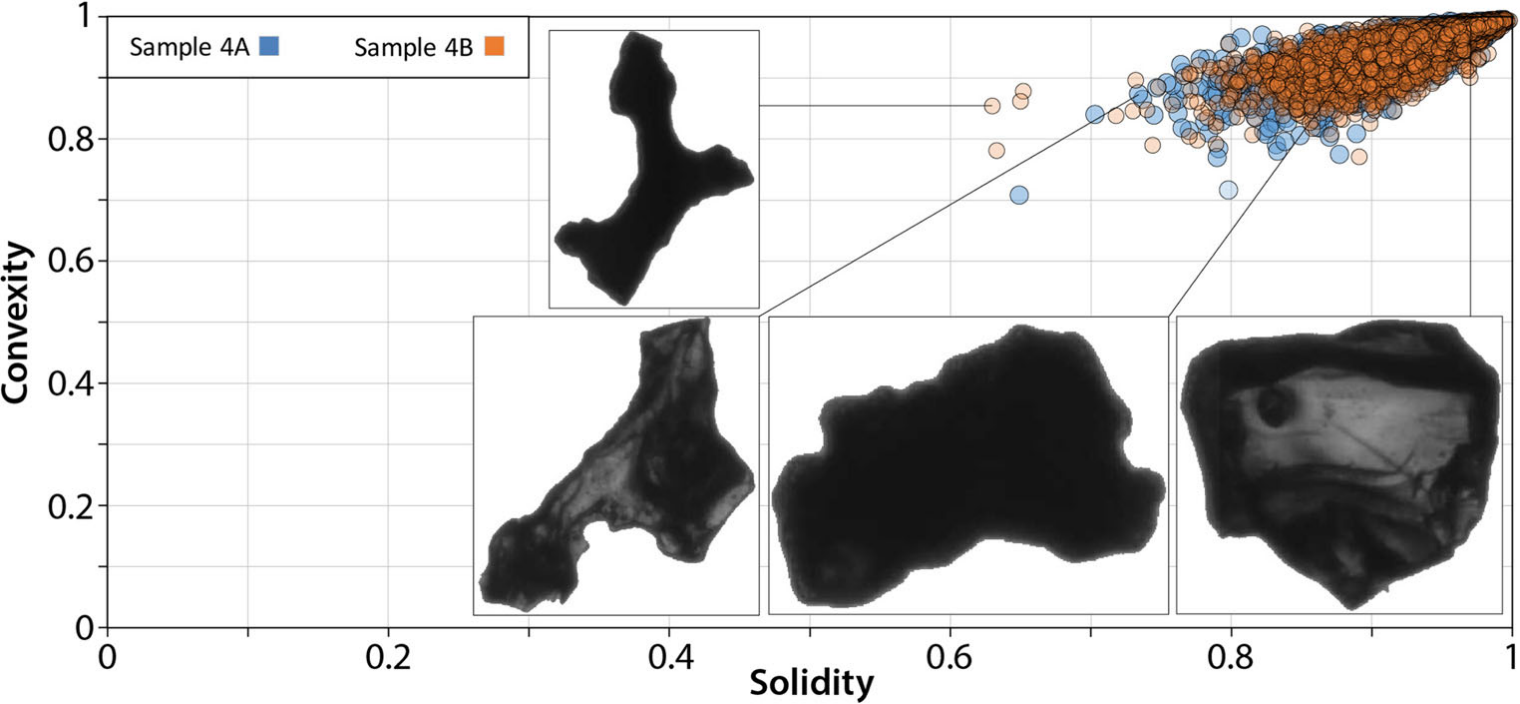
Tephra morphology measurements, representing convexity vs. solidity parameters. Analysis conducted at LMV-UCA

Tephra morphology measurements (Fig. 10) reflect the coexistence of both irregular (glassy and transparent textures) and blocky (microlite-rich and opaque textures) juvenile particles, as well as blocky crystals (conchoidal fractures and transparent textures).

**Fig. 11:**
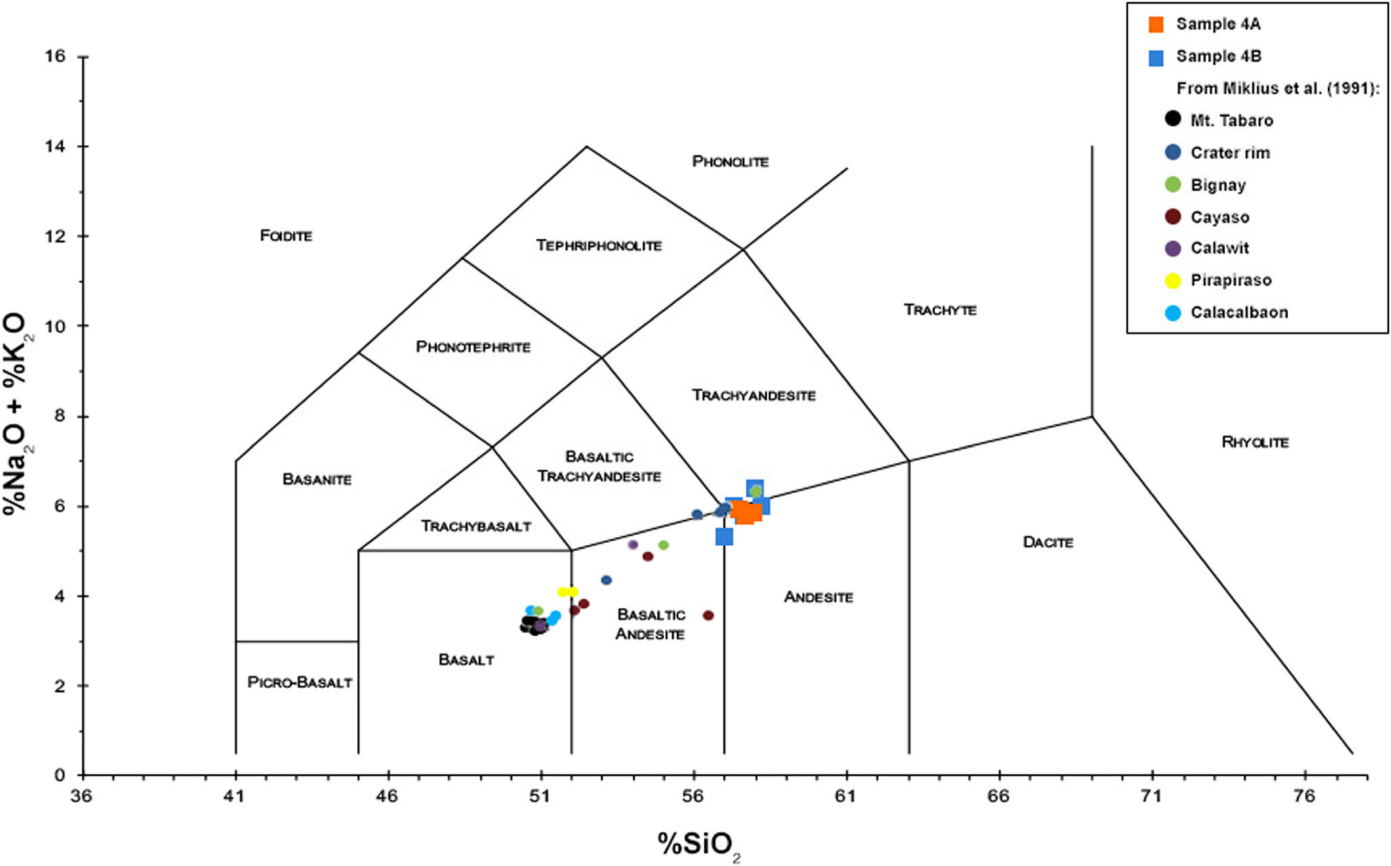
Plot of the geochemical analysis in this work against the whole-rock geochemistry of lava flow deposits and loose rocks from previous eruptions of Taal Volcano (Miklius et al. 1991). In situ glass compositions measured on juvenile microlite-free particles show an alkali-rich andesitic composition in the TAS diagram

In situ glass compositions measured on juvenile microlite-free tephra particles reveal that the erupted magma is andesitic in composition, with no significant variations within the two samples from Taal Volcano’s 12 January to early morning of 13 January eruption (Fig. 11).

## Discussion

### Analysis of isopach and isopleth data

The tephra fall of Taal Volcano during its 2020 unrest was dispersed over a large region covering an approximate area of at least 8605 km^2^. In a tropical environment where rainfall is common, tephra deposits can be easily washed away and deliberately removed by residents, forever erasing their depositional record. Using combined crowdsourcing the day after the eruption and field surveys within weeks after the event, tephra fall deposits were systematically documented, revealing some insights on the characteristics of the 12 January to early morning of 13 January eruption of Taal Volcano. Ashfall deposit measurement was found to be more accurate on flat, horizontal, and undisturbed surfaces. Field sampling focused on such areas and would make better observation points for netizens in future implementations of crowdsourcing ashfall data.

Using *TephraFits* to determine the thickness half-distance of tephra fall deposits, we calculate 1.40 km and 9.49 km for the proximal and distal exponential segments, respectively. The total calculated volume of erupted tephra for the 12 January to early morning of 13 January main eruption is 0.057 km^3^ (exponential model), 0.042 km^3^ (power-law model), and 0.090 km^3^ (Weibull model). All these computed values translate to a VEI of 3.

However, using the probabilistic approach (Weibull method) with 90% confidence interval, the volume estimate can be as high as 0.097 km^3^ which is close to the lower end of the volume range of VEI 4 (0.1–1 km^3^). Adding the volume of the base surge deposits amounting to 0.019 km^3^ (Lagmay et al. 2021), the total erupted volume translates to a VEI of 4, consistent with the classification criteria of Constantinescu et al. (2021), namely (1) volume of ejecta; (2) eruption plume height; and (3) umbrella cloud radius. The total erupted volume was calculated using crowdsourced and field survey data, whereas the erupted height and umbrella cloud radius are direct measurements from satellite data of Taal Volcano’s 2020 main eruption plume (Bachmeier 2020; Perttu et al. 2020; Lagmay et al. 2021).

Discrepancies between the power-law, exponential, and Weibull volumes which lead to different estimates of VEI values have been documented for other volcanoes. For example, in Layer 5 of the Cotopaxi (1180 ± 80 years B.P.) fall deposits, both power-law and exponential methods yielded a VEI 4 whereas the Weibull method resulted in a VEI 5 estimate (Biass et al. 2019). For Taal, all methods have a resulting volume corresponding to a VEI 3. A VEI 4 volume can be reached if the largest end of the largest estimate (Weibull method) is used with the addition of the base surge deposits. This is more consistent with the VEI classification of Constantinescu et al. (2021) for the observed plume height of 17–21 km (Bachmeier 2020; Perttu et al. 2020) and umbrella radius of 100 km (Lagmay et al. 2021) of Taal Volcano’s main eruption in 2020.

An eruption size at the low end of a VEI 4 perhaps explains the smaller volume and limited distribution of base surge deposits. Based on co-eruptive modeling of Bato et al. (2021), the magma reservoir at $\sim $5–6 km depth below TVI lost an estimated volume of −0.531 ± 0.004 km^3^. Much of this withdrawn magma is believed to have stalled at depth and emplaced as a northeast-striking dike extending under the towns of Taal, Lemery, Agoncillo, and San Nicolas, Batangas (Bato et al. 2021). Had most of the magma withdrawn from the reservoir been erupted in the 2020 event, it would still be classified as a VEI 4 with a more extensive pyroclastic surge deposit.

Maximum particle size data of the tephra deposits suggest that the 2020 eruption was larger than a “strong to moderate” event classified by PHIVOLCS at the time of eruption. We modeled an eruption height of 17.8 km using the Weibull method of *TephraFits*, which is consistent with the range of maximum heights measured from satellite images by Perttu et al. (2020) (16–17 km) and Bachmeier (2020) (20–21 km). Typical uncertainties of 20*%* for determining plume heights (Bonadonna and Costa 2013) yield a maximum value of 21.26 km. This calculation matches the above-anvil cirrus plume temperature measurement of $\sim 60^{\underline {\circ }}$C, which translates to approximately 21 km based on comparison of data with 3 rawinsonde sites in Legaspi, Mactan, and Laoag, Philippines (Bachmeier 2020). The calculations of thickness half-distance b_*t*_ and the ratio of thickness half-distance over thickness half-clast ($\frac {b_{t}}{b_{c}}$) plot in the sub-plinian classification scheme of Pyle (1989). A sub-plinian classification was also calculated using the classification scheme of Bonadonna and Costa (2013).

### Grain size distribution, morphology, and composition

**Fig. 12:**
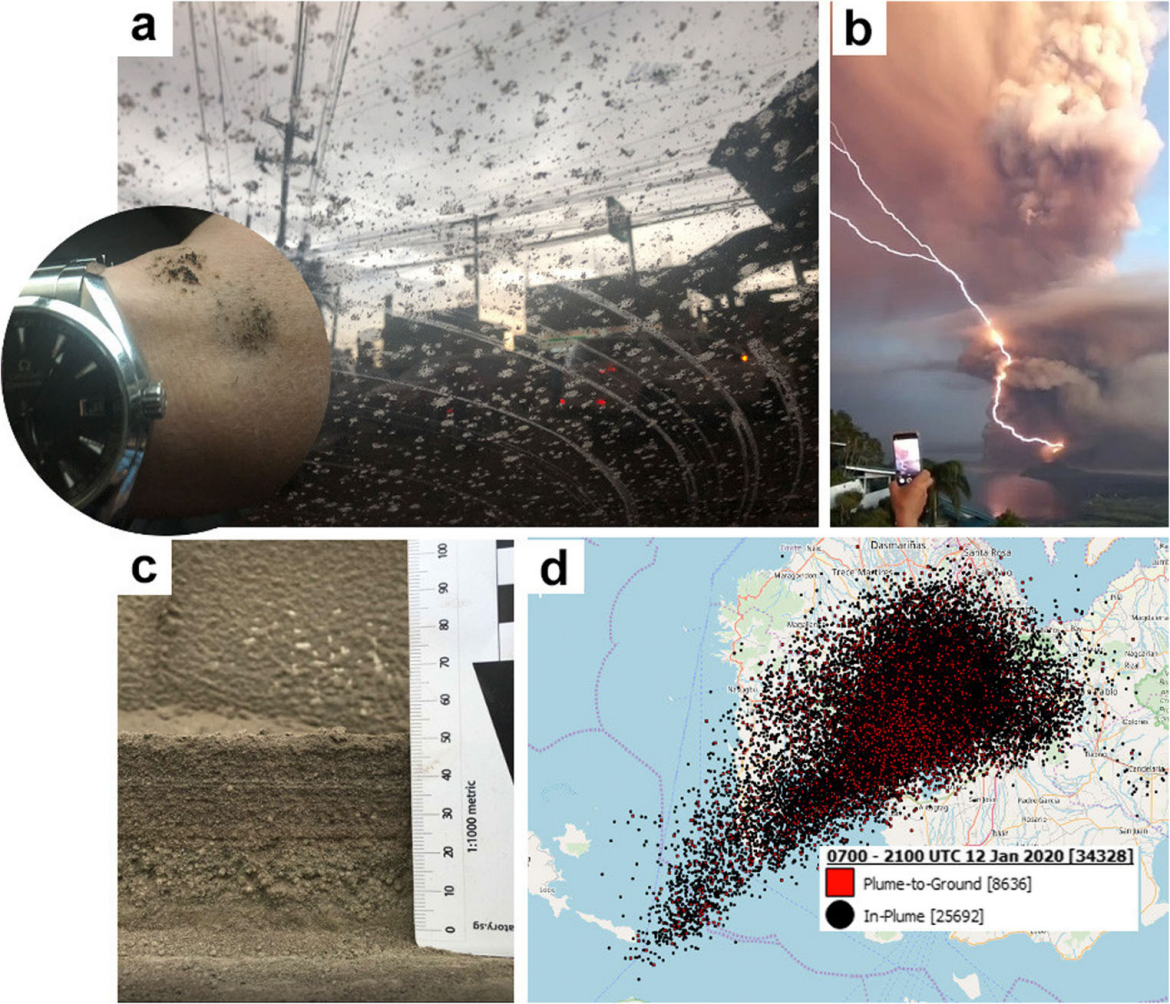
Evidence for a wet tephra plume. (a) Tephra falling in clumps on the car windshield in Silang, Cavite, 22 km north of Taal Volcano (inset shows clumps of tephra falling on the arm of author at 5:30 p.m. on 12 January). (b) Lightning during the eruption CC-BY Wikicommons. (c) Accretionary lapilli in tephra fall deposit. (d) Lightning produced during the eruption of Taal Volcano between 3:00 p.m. on 12 January and 5:00 a.m. on 13 January local time (0700–2100 UTC on 12 January). Red squares indicate plume-to-ground strokes; black circles indicate in-plume pulses. Courtesy of Chris Vagasky, Vaisala (Global Volcanism Program 2020)

The volcanic glass component of the tephra deposits represents newly erupted magma that came into contact with the MCL. Recent studies suggest that explosive magma-water interactions can be identified in some tephra features, such as grain size, componentry, morphology, and texture (e.g., Wohletz (2013), Jordan et al. (2014), Thivet et al. (2020), and Ross et al. (2021)). Nevertheless, phreatomagmatism fragmentation is still difficult to assess without any direct observation of the eruption itself (White and Valentine 2016). Present knowledge and monitoring of the Taal Volcano undoubtedly confirm the presence of a water lake (MCL) within the 2020 eruptive site. We also suggest that magma-water interaction favor (1) the fragmentation of some lithic grains (10–17% of the counted grains), (2) the occurrence of specific textural signatures (ubiquitous hackle lines, stepped features, and micro-fractures), and (3) the relatively fine grain size of the deposit (grain modes ranging between 90 and 500 μm depending on the deposit locations). Lithic grains, including hydrothermally altered fragments, sub-rounded volcanic rock fragments, and oxidized grains are those plucked from the volcano’s conduit during the eruption. Free crystals found in the tephra fall deposit were formed earlier in the magma body’s ascent history and liberated from the glassy matrix during eruption fragmentation. A phreatomagmatic eruption starting at about 4:00–5:30 p.m. of 12 January resulted in wet tephra to fall in clumps (Fig. 12). The excess water included in the eruption may have contributed to the dramatic display of thousands of lightning strokes and bolts (Prata et al. 2020; Van Eaton et al. 2020) (Fig. 12b) throughout the period of the sub-plinian eruption.

Accretionary lapilli were abundant in the tephra fall deposits, especially in the areas of Agoncillo, Laurel, and San Nicolas Batangas (8–9 km away). In more distal areas, including Metro Manila (65 km away), tephra fall deposits include irregular-shaped aggregates with no specific internal grain organization. The “wet” nature of the eruption can be attributed to the interaction of the eruptive materials with the 42 million m^3^ of water contained in the MCL (Bernard A et al. 2020) as evidenced by lapilli-sized aggregates. This is consistent with the interpretation of a phreatomagmatic eruption starting at about 4:00–5:30 p.m. of 12 January, which resulted in wet tephra to fall in clumps (Fig. 12). Base surges, known to form from the interaction of magma and water, are also reported to have cascaded down the slopes of Taal Volcano. They may have travelled across the lake up to about 600 m beyond the island’s shores based on the energy-line model (Lagmay et al. 2021).

### Composition of juvenile fragments

The glass fraction of juvenile tephra is andesitic in composition for the two samples that were analyzed. The whole-rock geochemistry of lava flows and loose rocks from the Volcano Island reported by Miklius et al. (1991) is plotted on the Le Maitre et al. (2005) TAS diagram showing most of the older eruption products were basalt to basaltic andesite in composition (Fig. 11). The 2020 microlite-free juvenile tephra materials are more andesitic and contain higher SiO_2_ (55–56%) and Na_2_O + K_2_O (< 5.5%) than most of the older volcanic rocks. As a preliminary interpretation, this suggests that the magma erupted during the 2020 eruption is slightly more evolved relative to the 1968–1969 lavas erupted from Mt Tabaro, located southwest of Taal Volcano Island. However, for loose samples collected from the Main Crater rim and lavas from the Bignay eruption center, the composition is quite similar to the 2020 tephra (Fig. 11). A more detailed study of the 2020 eruptive products is the topic of another paper in preparation that will compare whole-rock geochemistry from different eruptive events.

### Tephra fall (ashfall) hazards

**Fig. 13:**
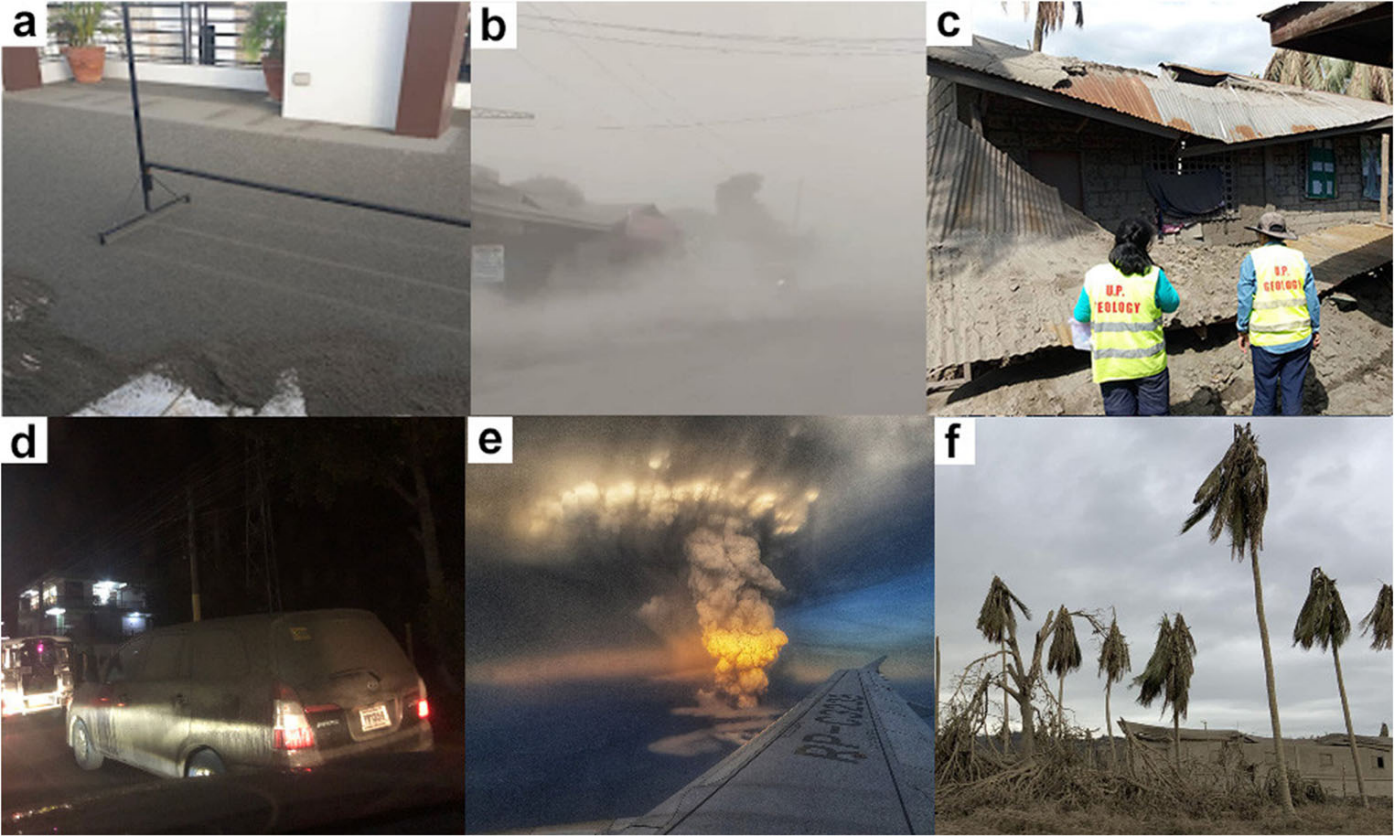
Tephra fall impacts. Ashfall (a) blanketing a garage in Laguna Province; (b) covering road surfaces; (c) causing the collapse of roofs; (d) impairing road visibility; (e) causing danger to inbound flights to Manila; and (f) damaging crops

The eruption of Taal Volcano deposited heavy, corrosive ash onto exposed surfaces such as house fittings (i.e., external pipes and gutters) and roads (Fig. 13a). The interior of buildings were also affected by wind-blown ash, causing massive disruption of services (e.g., suspending work and school) and posing hazards to the health of residents (Fig. 13b) (Baxter et al. 1982). In more severe cases, such as in the municipalities of Agoncillo and Laurel in Batangas, houses made of light materials collapsed as wet ash increased the load on roofs and walls (Fig. 13c).

Ground transportation was heavily impacted as primary and resuspended ash dispersed in air and caused reduced visibility and traction of road networks (Fig. 13d). To wash off ashfall, cars needed to automatically spray water while wiping. Without spraying water, ash remained on the windshield and scratched the glass, reducing visibility for the car driver. The water tank for the wiper spray needed to be replenished after only a few minutes. For aviation, several airports including the Ninoy Aquino International Airport (NAIA) had to suspend over 240 flights (Rappler 2020; Reuters 2020; Chen 2020) as flying through ash clouds (Fig. 13e) is known to cause significant damage to aircraft engines (Casadevall 1992; Guffanti et al. 2010).

Agriculture areas were also damaged by extensive tephra fall. Within the municipality of Agoncillo in Batangas, the leaves of coconut trees sagged and were on occasion felled by ashfall (Fig. 13f). Pineapple crops and trees in Tagaytay, Cavite, were blanketed by ash for weeks. Taal Volcano’s eruption caused severe damage to the Philippines’ agricultural sector, with losses climbing to PhP 3.06 billion (USD 59.98 million/ EUR 54.19 million) according to the Department of Agriculture (CNN Philippines Staff 2020). Affected were coffee, cacao, pineapple, rice, coconut, and other high-value produce. The fisheries sector recorded the highest value of damage after the eruption, as it lost PhP 1.6 billion (USD 31.36 million/EUR 28.33 million) for the tilapia and milkfish (bangus) culture around the Taal Lake (CNN Philippines Staff 2020).

The National Disaster Risk Reduction and Management Council (NDRRMC) ordered the evacuation of at least 18,187 residents and housed them in 76 evacuation centers (MMDA 2020) because of the threats posed by the eruption and from ashfall. People were advised by the Department of Health (DOH) to stay indoors and wear medical masks to avoid inhalation of ash particles and skin exposure (Viray 2020).

## Conclusions

On 12 January 2020, Taal Volcano erupted after 43 years of dormancy. Within a few hours from the start of the event at about 1 p.m., a towering 17–21-km-high eruption plume formed with its umbrella cloud drifting towards the north–northeast, affecting nearby provinces, including Metro Manila, the National Capital Region of the Philippines. Tephra fall deposits mantled the ground surface, covering an area of 8605 km^2^ as far as 70 km north–northeast of Taal Volcano.

Tephra fall deposits were mapped using crowdsourced data collected during the 12–13 January main eruption, and field surveys within weeks after the peak eruptive period. This complementary technique for gathering data is particularly useful for a tropical country where tephra fall deposits are easily washed away by rain.

The *TephraFits* model results show that the tephra fall thins downwind exponentially with a thickness half-distance of about 1.40 km and 9.49 km for the proximal and distal exponential segments, respectively. In terms of tephra fall volume, model results using the exponential, power-law, and Weibull models, yield values of 0.057 km^3^, 0.042 km^3^, and 0.090 km^3^, respectively; all of these values translate to a VEI of 3.

However, using the probabilistic approach (Weibull method) with 90% confidence interval, the volume estimate can be as high as 0.097 km^3^. With the addition of the base surge deposits of 0.019 km^3^ (Lagmay et al. 2021), the total eruption volume translates to a VEI of 4, which is more consistent with the classification for the observed plume height of 17–21 km (Perttu et al. 2020; Bachmeier 2020) and umbrella radius of Taal Volcano’s main eruption in 2020. A VEI of 4 is also consistent with the calculated median height of 17.8 km and sub-plinian classification which are based on the combined analysis of isopleth and isopach data derived from crowdsourced and field data.

The eruptive vent, located at the MCL, and the numerous fissures generated by ground deformation, allowed the explosive interaction of magma and water. Analyses of the fall deposits using binocular and electron microscopes reveal vitric (83–90%), lithic (7–11%), and crystal components (< 6%) that indicate a phreatomagmatic eruption. This interpretation is supported by the presence of accretionary lapilli in fall deposit layers and tephra falling in wet clumps at the height of the eruptive event. Textural signatures on juvenile particles, such as stepped features, also support the occurrence of the interaction between magma and external fluids at the fragmentation level, which enhanced the formation of fine ash particles.

Although the primary eruption lasted for only about 10 h, the damage to infrastructure and crops, and effects on air quality were significant. Eruptive activity continued for 7 days but was mainly characterized by discrete cannon-like explosions that generated 2-km-high bent-over plumes on 13 January that drifted to the west and southwest. Waning of activity generated much lower eruption heights of about 500 m until the eruptive activity ended on 22 January (PHIVOLCS 2020a; 2020b).

This study is a culmination not only of the authors’ own work, but also of the contributions of hundreds of members of the general public who stepped up to the call of citizen science, providing invaluable data and making themselves part of this crucial scientific work. Through their work and our own, we advance the knowledge on Taal Volcano, the second most active eruptive center in the Philippines with a long history of devastating eruptions. The growing density of population and rapid development of areas surrounding Taal Volcano make such understanding of its consequent tephra fall hazards and their impacts more significant.

## Electronic supplementary material

Below is the link to the electronic supplementary material.

(MOV 21.8 MB)
